# Category Name Expansion and an Enhanced Multimodal Fusion Framework for Few-Shot Learning

**DOI:** 10.3390/e27090991

**Published:** 2025-09-22

**Authors:** Tianlei Gao, Lei Lyu, Xiaoyun Xie, Nuo Wei, Yushui Geng, Minglei Shu

**Affiliations:** 1 The College of Computer Science and Engineering, Shandong University of Science and Technology, Qingdao 266590, China; gaotl@sdas.org; 2 Shandong Artificial Intelligence Institute, Qilu University of Technology (Shandong Academy of Sciences), Jinan 250014, China; xiexy@sdas.org (X.X.); wein@sdas.org (N.W.); 3 School of Mathematics and Statistics, Qilu University of Technology (Shandong Academy of Sciences), Jinan 250014, China; 4 The School of Information Science and Engineering, Shandong Normal University, Jinan 250014, China; lvlei@sdnu.edu.cn

**Keywords:** category name expansion, image feature augmentation, cross-modal residual connection, multimodal fusion, semantic representation

## Abstract

With the advancement of image processing techniques, few-shot learning (FSL) has gradually become a key approach to addressing the problem of data scarcity. However, existing FSL methods often rely on unimodal information under limited sample conditions, making it difficult to capture fine-grained differences between categories. To address this issue, we propose a multimodal few-shot learning method based on category name expansion and image feature enhancement. By integrating the expanded category text with image features, the proposed method enriches the semantic representation of categories and enhances the model’s sensitivity to detailed features. To further improve the quality of cross-modal information transfer, we introduce a cross-modal residual connection strategy that aligns features across layers through progressive fusion. This approach enables the fused representations to maximize mutual information while reducing redundancy, effectively alleviating the information bottleneck caused by uneven entropy distribution between modalities and enhancing the model’s generalization ability. Experimental results demonstrate that our method achieves superior performance on both natural image datasets (CIFAR-FS and FC100) and a medical image dataset.

## 1. Introduction

With the rapid advancement of image processing technology, the cost of acquiring annotated data has gradually increased, especially in medical imaging and certain specific natural image domains. This makes building large-scale training datasets extremely difficult. To address this challenge, few-shot learning (FSL) has garnered increasing attention [1,2,3,4]. The goal of few-shot learning is to acquire sufficient knowledge from a limited number of annotated samples and effectively apply it to tasks such as classification and detection [5,6].

From an information-theoretic perspective, the core dilemma of few-shot learning lies in the challenge of information entropy. When the number of samples is scarce, the amount of information that each category can provide is extremely limited. This means that our understanding of a category’s true data distribution is highly uncertain, i.e., the prior entropy of that category is very high. Although traditional few-shot learning methods can learn effectively with data scarcity, this field still faces many challenges, particularly in improving the model’s generalization ability to new samples and avoiding performance degradation caused by sample scarcity.

Traditional few-shot learning methods typically rely on metric learning, where both the support set and query set are mapped into a metric space, and classification is performed using nearest neighbor search. However, the effectiveness of these methods is often limited by the quantity and quality of samples in the support set. Due to sample scarcity, the support set often cannot fully capture the diversity within a category. This means that the amount of information provided by the support set is insufficient to significantly reduce the uncertainty of the category distribution. This leads to unstable model performance when encountering new, unseen samples. Especially when support samples are located at the edges of the metric space, it is difficult for the model to extract sufficiently discriminative features from these scarce samples, thereby affecting the accuracy and stability of classification.

With the continuous development of deep learning, utilizing visual features for classification or recognition has achieved some success in certain tasks. However, when faced with limited annotated samples, single-modal models expose some obvious shortcomings. A core challenge of few-shot learning is understanding how to improve a model’s generalization ability to unseen samples. Relying solely on visual features, especially models trained from a single modality, may introduce bias when processing unseen samples. Due to limited training samples, the model struggles to learn comprehensive representative features, which makes it difficult to handle new, unseen categories or variations, thus affecting generalization performance. This is essentially because the information available from a single source (visual modality) to reduce classification uncertainty is limited.

To address this problem, few-shot learning research combining multimodal features like text and images has gradually attracted attention [7]. However, most existing multimodal few-shot methods heavily rely on category names or extended category names as textual input. While this approach can enhance the model’s recognition capability to some extent, it still has certain limitations. Firstly, category names and their extensions often rely on fixed textual descriptions or synonyms, which may not fully capture the detailed features in an image. For example, in medical image analysis, different manifestations of lesions or individual patient variations are often difficult to fully describe with simplified category names. Secondly, pure text enhancement cannot reflect visual features such as spatial information, texture, and shape within the image, which are crucial for accurate classification. From an information-theoretic perspective, category names themselves are low-entropy information, and the discriminative information they carry is insufficient, so simply extending the names cannot effectively improve model performance under few-shot conditions. In this context, cross-modal residual connections play an important role, as they enable complementary information from both modalities to be adaptively integrated while preserving the core representations of each. Such connections allow textual cues to refine visual features without overwhelming them, leading to more balanced multimodal representations. However, most existing residual designs in multimodal learning remain relatively shallow or linear, which may result in redundant information flow and limit the exploitation of deeper cross-modal interactions. Therefore, it is necessary to revisit and refine the design of cross-modal residual connections to better address the challenges of few-shot learning.

To address the aforementioned challenges, this paper proposes a novel method based on category name extension, which incorporates sample-specific image features as additional information. Unlike traditional methods that rely solely on textual information for category extension, our method introduces visual modality features by combining the visual characteristics of sample images with category text. This fusion process enhances the semantic representation of each category and increases the information entropy of category embeddings, enabling them to capture more diverse and information-rich cues. From an information-theoretic perspective, the enrichment of entropy reflects a greater ability to describe the inherent variability within a category. By jointly modeling category names and image features, the proposed method allows the model to capture fine-grained visual differences beyond just category labels. Consequently, each category representation not only encompasses semantically meaningful descriptions but also integrates structural and textural information extracted from real images, effectively compensating for the limitations of pure text enhancement in complex tasks and improving the model’s sensitivity to subtle visual details.

Furthermore, to obtain higher-quality multimodal fusion features, we propose an improved feature fusion strategy aimed at optimizing the joint representation of image and text modalities. In traditional multimodal learning frameworks, visual and textual information are typically fused through simple concatenation or weighted averaging. Although these methods allow for basic information transfer between modalities, their fusion architecture lacks a conscious, entropy-guided regulation mechanism, making it difficult to effectively balance redundant and useful features. This can lead to the proliferation of irrelevant information or the dilution of crucial signals. To address this problem, we introduce an attention-based feature fusion strategy that includes a dynamic weighting mechanism. This allows the model to adaptively assign importance to different modalities based on the task context and the uncertainty of the input data. The main contributions of this paper are as follows:

- The combination of category name expansion and image feature augmentation: Based on traditional category name expansion, we incorporate specific sample image features to supplement the expanded content. By introducing image features, the semantic description of the categories becomes more precise and detailed, effectively improving the performance of multimodal few-shot learning.
- Improved visual and textual feature fusion method: In each layer of feature fusion, we designed a cross-modal residual connection structure to ensure that the output at each layer retains the original information while facilitating the effective alignment of multimodal information.
- Multi-task validation: We validated the proposed method’s effectiveness across multiple few-shot learning tasks. Experimental results demonstrate that our approach outperforms existing multimodal few-shot learning methods across various datasets, showcasing its broad applicability and excellent performance in different domains and tasks.

## 2. Related Works

### 2.1. Few-Shot Learning

Few-shot learning aims to improve model generalization under conditions of limited samples by leveraging prior knowledge or developing effective methods. Research in this field primarily focuses on meta-learning-based methods, transfer-learning-based methods, data augmentation-based methods, and metric-learning-based methods.

Meta-learning [8,9,10] is a method that focuses on developing a generalizable learning strategy across multiple tasks. It can be divided into three main types. Optimization-based meta-learning adjusts the initial parameters of the model so that it can achieve good performance on new tasks with minimal gradient updates. Andrychowicz et al. [11] treated the design of optimization algorithms as a learning problem, allowing LSTM-based learning algorithms to outperform manually designed ones and generalize effectively to similar tasks. Memory-based meta-learning [12] incorporates external memory modules to store shared information, enabling the model to adapt quickly to new tasks. Task-distribution-based meta-learning involves calculating class prototypes (mean vectors of support samples) and classifying based on nearest-neighbor principles, enhancing few-shot learning outcomes.

Transfer learning addresses the issue of data scarcity by transferring knowledge from large-scale pre-trained models to small sample tasks. Data augmentation alleviates the problem of limited data by generating or expanding samples, thus enhancing model generalization. For example, RBC [13], based on transfer learning using AlexNet feature extraction and optimized CNN classification, improves accuracy compared to existing methods.

Metric learning [14,15,16,17,18,19,20] focuses on designing an efficient embedding space where the model can directly perform classification or regression by learning task-relevant similarity metrics. For example, Cross-transformers [21] uses a distance-based metric to infer category membership by calculating the distance between labeled images and unlabeled query images in the feature space. Simple CNAPS [22] introduces class covariance distance metrics (i.e., Mahalanobis distance) into the CNAPS framework, significantly enhancing performance. It demonstrates the ability to estimate high-dimensional feature covariance from very few samples using an adaptive feature extractor. Specifically, the model optimizes the relative distances between samples to ensure that similar samples cluster together while samples from different categories are separated in the embedding space.

Although current methods have made progress in small-sample tasks, most rely on assumptions of task distribution consistency or have high computational costs, particularly in multiple optimization updates. Traditional approaches mainly focus on single-modality tasks and lack adaptability in multimodal scenarios, such as joint reasoning between images and text. Therefore, multimodal few-shot learning has become a new research direction, emphasizing how to leverage multimodal information to collaboratively enhance the model’s generalization ability.

### 2.2. Multimodal Few-Shot Learning

Multimodal few-shot learning has become a key research area in fields like computer vision and natural language processing. It aims to enhance model generalization by integrating information from different modalities (such as images, text, speech) when labeled data is scarce. Zhang et al. [23] proposed semantic evolution, an approach to automatically generate high-quality semantics to address challenges in few-shot learning. By introducing these semantics, the framework avoids the complex networks and algorithms used in previous methods. Multimodal MAML [24], by learning a shared strategy across multiple tasks, enables the model to quickly adapt to new multimodal tasks with only a few samples.

In addition, transfer learning has also made significant progress in multimodal few-shot learning. Models like AMU-Tuning [25] learn joint representations of images and text through large-scale pretraining on multimodal data, enabling them to perform well in few-shot tasks. On the other hand, multimodal learning methods based on attention mechanisms, such as Cross-Attention Networks (CANs) [26], improve cross-modal fusion by adaptively weighting different modalities. Furthermore, generative models like CycleGAN [27] and VAE [28] are used to augment datasets by generating new samples, addressing data scarcity and enhancing learning ability in few-shot conditions. Finally, cross-modal contrastive learning maximizes the similarity and minimizes the differences between samples from different modalities, further improving cross-modal learning capability. MSQIF [29] reexamines QBBA entropy to investigate the QBBA negation process and designs several multi-source quantum information fusion algorithms to support decision-making, which are further applied to pattern classification. CECC-WDMSIF [30] designs a weighted-discount multi-source information fusion algorithm based on the complex evidence correlation coefficient to improve the performance of expert systems. Despite these advances, multimodal few-shot learning still faces many challenges, such as how to effectively fuse different modalities, design more powerful cross-modal generative models, and improve model interpretability. These remain important directions for future research.

## 3. Method

### 3.1. Problem Description

Few-shot learning tasks typically involve two datasets: the Base Set and the Novel Set. The Base Set is used to train the model’s metric space, while the Novel Set is used to test the model’s performance on unseen categories. Specifically, the Base Set *D*_*base*_ and the Novel Set *D*_*novel*_ can be defined as follows:

(1) $$D_{base}=\left\{\left(x,y\right)|x\in X_{base},y\in C_{base}\right\},D_{novel}=\left\{\left(x,y\right)|x\in X_{novel},y\in C_{novel}\right\},$$

where *x* represents a sample, *y* represents the label, and *C*_*base*_ and *C*_*novel*_ are the category sets of the Base Set and the Novel Set, respectively. Moreover, *C*_*base*_ ∈ *C*_*novel*_ = ∅, where there is no overlap between the label spaces of the two sets.

In the training phase, the Base Set *C*_*base*_ is used to learn a feature embedding function $f_{\theta }:\chi \to R^{d}$ that maps each sample *x* to a *d*-dimensional metric space. The Base Set contains a large number of categories and samples, $\left|C_{base}\right|\gg \left|C_{novel}\right|$, where $\left|\cdot \right|$ denotes set cardinality. The model is trained to maximize the mutual information *I*(*Z*;*Y*) between the embedded features *Z* = *f*_*θ*_(*X*_*base*_) and the category labels *Y*, which encourages the model to be sensitive to category-specific features:

(2) $$\theta^{*}=arg\underset{\theta }{max}I\left(f_{\theta }\left(X_{base};Y_{base}\right)\right).$$

This training can be interpreted as modeling the category distribution *p*(*y* | *x*) under a high-entropy condition to enhance generalization to unseen categories. By maximizing the mutual information between the support samples and the category distribution, the model enhances its sensitivity to category-specific features, thereby facilitating effective knowledge transfer.

In the testing phase of few-shot learning, the Novel Set *D*_*Novel*_ can be further divided into a Support Set and a Query Set. Specifically, the Support Set *S* contains *K* samples from each of the *N* categories in the Novel Set *D*_*Novel*_, which are used to train the model. The design of the support set enables the model to convey as much category information as possible using only a limited number of samples. The amount of information carried by the support set can be interpreted as the model’s initial conditional entropy within the new category space. The Query Set *Q* contains *M* query samples related to the categories in the Support Set *S*, and these samples are used to evaluate the model’s classification performance. The relationship between the Support Set and the Query Set can be expressed by the following formula:

(3) $$S={\left\{(x_{i},y_{i})\right\}}_{i=1}^{N\times K},\;Q={\left\{(x_{i},y_{i})\right\}}_{i=1}^{N\times M},$$

where *x*_*i*_ represents the sample, *y*_*i*_ represents the corresponding label, *N* is the number of categories in the Support Set, *K* is the number of labeled samples per category, and *M* is the number of query samples in the Query Set. In FSL tasks, the *N*-way *K*-shot setting is often used to describe the number of categories and samples in the task. For example, assuming *N* = 5 and *K* = 1, it means the Support Set contains 5 categories, with 1 sample per category. This setting implies that the model needs to learn how to classify effectively from a small number of labeled samples.

To achieve the above goal, we propose a multimodal few-shot learning approach based on category name expansion and image feature supplementation. In the following sections, we will introduce our method in terms of three aspects: First, we provide an overview of the model architecture and core design. Next, we analyze the training process, including how the Base Set is utilized to enhance generalization. Finally, we explain the testing phase in detail, focusing on the roles of the novel support and query sets and their application in few-shot learning.

### 3.2. Overall Architecture of the Model

**Figure 1:**
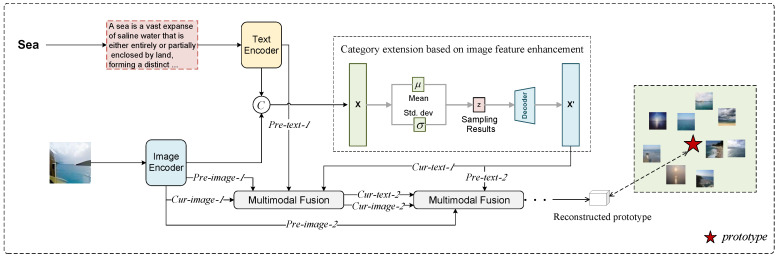
Flowchart of the Training Phase. After category names are expanded, they are input into their respective encoder along with images. The outputs are concatenated, and the image features are used to augment the text features. The fusion process explicitly integrates the encoder-derived features with the enhanced representations and is implemented in a multi-layer stacked manner to progressively refine cross-modal interactions.

In this section, we provide a detailed introduction to the proposed multimodal few-shot classification model. The model integrates category name expansion with image feature supplementation to enhance the expressiveness of the textual modality. Additionally, a multi-stage fusion strategy is designed to optimize category prototype representations, thereby improving the performance of multimodal few-shot classification tasks. The training-phase diagram shown in Figure 1 illustrates the overall architecture of the model.

First, category names are expanded and processed by a text encoder to extract initial text features. Simultaneously, images are processed through an image encoder to obtain visual features. The extracted text and image features are then concatenated and fed into a multi-level fusion module. This module integrates category semantics with structural and textural information from the visual modality, thereby increasing the information entropy of the category representations. This enhancement helps mitigate information loss and semantic ambiguity caused by reliance on a single modality.

Next, the enhanced text features and image features are further processed by the multimodal fusion module, which adopts a multi-stage fusion strategy. In the first stage, the initial text and image features serve as the pre-stage features. These initial features provide the original unimodal semantics, preventing semantic drift after multiple stages of fusion. At the same time, they continuously participate in the multi-stage fusion process, offering contextual support to the current stage and ensuring that the model can still capture key semantics under data-scarce few-shot conditions. The enhanced text features and the initial image features are used as the current-stage features for fusion. In the second stage, the enhanced text features and initial image features serve as pre-stage features, with the fusion results from the previous stage used as current-stage features for further integration. The multi-stage fusion not only enables progressive alignment and complementation of features across modalities but also introduces new information flow that gradually reduces the conditional entropy of category representations, enhancing the model’s ability to distinguish subtle differences between categories. Through multiple stages of fusion, the final multimodal representation is obtained. The fused features are then used to construct class prototypes, enabling similarity matching in the feature space for precise few-shot classification.

This section is divided into two parts: first, we introduce the multi-level fusion module for category name expansion and image feature supplementation; then, we detail the prototype construction process based on the multi-stage fusion module.

#### 3.2.1. Multi-Level Fusion Module for Name Expansion and Image Feature Augmentation

In multimodal few-shot classification tasks, the textual modality serves as one of the key information sources and typically relies on category names for classification. However, the expressiveness of category names is inherently limited and often fails to capture the full semantic scope of a category. Recent studies have attempted to enhance category understanding by leveraging large language models (LLMs) to expand category names into richer textual descriptions. While this approach can increase the information content of the textual modality—effectively raising the semantic entropy of category representations—it often depends on predefined vocabularies or synonyms and related expressions generated by the model, which may not fully capture the visual characteristics of the category. To address this issue, we propose integrating image features with category text expansion. As a high-dimensional perceptual modality, image features inherently encode low-entropy representations of category-specific information such as spatial structures, textures, and shapes. These features can effectively supplement the fine-grained details that are often missing from purely language-based descriptions. By incorporating image features into the text expansion process, we aim to increase the mutual information between the generated text and visual content, thereby enhancing the alignment between modalities. This strategy improves the accuracy and consistency of category representations and ultimately boosts model performance and generalization in few-shot classification tasks.

As described in Figure 1 for this section, first, given a category *t*, we obtain an initial category name text *C*_*t*_. Inherently, a simple category name (for instance, ‘cat’) possesses high entropy, corresponding to a large degree of semantic ambiguity. To reduce this ambiguity and enrich the information content, we leverage a large language model to expand upon the category name, utilizing the same prompts as in SemFew [23] to generate a more descriptive text $C_{t}^{expand}$. Input it into a text encoder (e.g., BERT) and take the output [CLS] token as the latent representation of the text:

(4) $$z_{t}^{text}=BERT\left(C_{t}^{expand}\right)\left[CLS\right],$$

where $z_{t}^{text}$ is the latent variable of the text, which includes its semantic information and encodes the text into a fixed-dimensional vector.

Next, based on the previous content, we utilize image data to enhance the contextual richness of text descriptions for each category *t*. Specifically, we have a set of image samples *S*_*t*_ representing the category *t*, where each image sample *x*_*i*_ belongs to the category *t*. The set of image samples can be represented as

(5) $$S_{t}={\left\{x_{i}\right\}}_{i=1}^{N_{t}},$$

where *N*_*t*_ represents the number of image samples for the category *t* and *x*_*i*_ denotes the *i*-th image sample in the category *t*. To enable the model to effectively leverage the visual features from the images, we employ feature extractors such as CNNs or ViTs to obtain image representations. The choice of feature extraction architecture follows two main criteria: (1) its proven ability to capture discriminative representations in few-shot learning tasks and (2) its compatibility with our training paradigm in terms of computational efficiency and scalability. Specifically, CNNs are adopted due to their strong capability in modeling local spatial patterns and low-level textures, which are crucial for medical and natural image analysis. In contrast, ViTs are utilized for their global receptive fields and self-attention mechanisms, enabling them to effectively capture long-range dependencies and semantic correlations:

(6) $$P_{t}^{image}=f\left(x_{i}\right),$$

where *f*(*x*_*i*_) is the feature vector extracted from the image sample *x*_*i*_ after processing by the CNN or ViT model, representing the visual information of the image. This feature extraction process acts as efficient information compression. The model *f*(*x*_*i*_) captures key visual information for category perception (like textures and outlines) from high-dimensional raw pixel data and filters out irrelevant noise. These features provide important contextual information for the text expansion process, helping to generate text expansion that is more visually consistent.

In a few-shot setting, representations from a single modality—whether text or images—contain limited information and thus possess high entropy (or uncertainty). Our strategy is designed to fuse these two complementary sources of information, thereby significantly lowering the uncertainty of the resulting joint representation. Specifically, we extract semantic latent variables $z_{t}^{text}$ corresponding to category names and integrate them with image features $P_{t}^{image}$ extracted from support samples of that category. Through fusing these dual-modality representations, we construct a joint representation $z_{t}^{joint}$ that simultaneously captures textual semantics and the visual context of categories. This fused representation significantly enhances discriminative power, providing the groundwork for generating more distinctive expanded category descriptions under few-shot conditions.

First, we concatenate the semantic latent variable $z_{t}^{text}$ extracted from category names with the visual features $P_{t}^{image}$ obtained from support set images. This concatenated representation is processed through a fusion layer to generate an enriched joint representation *z*_*t*_:

(7) $$z_{t}=f_{m}\left(concat\left(z_{t}^{text},P_{t}^{image}\right)\right),$$

where *f*_*m*_ denotes a fully connected mapping layer that projects the fused multimodal features into a shared latent space. In few-shot scenarios, single-modality representations (e.g., text alone) exhibit limited expressive power. By integrating semantic and visual information, this step constructs more comprehensive category representations, effectively mitigating feature instability issues under data scarcity conditions.

Secondly, to further enhance the robustness and generalization of category representations in multimodal few-shot classification, we introduce a distribution modeling mechanism aimed at capturing the potential semantic and visual variability within each category. Our core insight is that when labeled samples are extremely scarce, relying on a single fixed vector to represent an entire category is essentially a ‘point estimate’. While this method achieves very high information compression, it consequently ignores the rich internal diversity of the category. Therefore, instead of representing a category as an isolated point, we model it as a probability distribution. This approach allows us to not only capture the category’s ‘central’ information (the distribution’s mean) but also to quantify its range of semantic and visual variation (the distribution’s variance).

Specifically, after obtaining the fused category representation *z*_*t*_ (integrating textual semantics and support image features), we avoid directly using it as the final category vector. Instead, we construct a learnable category embedding distribution modeled $z_{t}^{joint}$ as a Gaussian:

(8) $$q\left(z_{t}^{joint}|C_{t}^{expand},P_{t}^{image}\right)=\Delta \left(\mu_{t}^{joint},\sum_{t}^{joint}\right),$$

where $\mu_{t}^{joint}$ and $\sum_{t}^{joint}$ are the mean and covariance matrix of the joint latent variable, respectively, which are learned from *z*_*t*_ through the fully connected layer. Supposing that we have the conditional information y, our goal is to approximate the true posterior distribution $p(z_{t}^{joint}|C_{t}^{expand},P_{t}^{image})$ using a variational distribution $q(z_{t}^{joint}|C_{t}^{expand},P_{t}^{image})$ and sampling from this distribution to obtain $z_{t}^{joint}$.

To sample the latent variable $z_{t}^{joint}$ from the latent space, we employ the reparameterization trick. This trick allows us to transform the sampling process of the Gaussian distribution into a deterministic one, enabling the entire generative process to be optimized via backpropagation. The reparameterization process can be written as

(9) $$z_{t}^{joint}=\mu_{i}^{joint}+\epsilon \sigma_{i}^{joint},$$

where *ϵ* ∈ (0, *I*) is noise sampled from a standard normal distribution, and $\sigma_{i}^{joint}$ is the standard deviation of the joint latent variable. In this context, the standard deviation $\sigma_{i}^{joint}$ is not merely a statistical parameter but serves as a direct measure of the information entropy for the category’s joint representation. This value quantifies the inherent uncertainty and variability within the representation that emerges from the fusion of text and visual information. By using the reparameterization trick, we can sample from the variational distribution to generate the latent variable $z_{t}^{joint}$. The joint latent variable $z_{t}^{joint}$ is generated by combining the text latent variable $z_{t}^{text}$ and image features $P_{t}^{image}$. As a result, the joint latent variable contains both the semantic information from the text and the visual information from the image. This information is then used by the decoder to generate more accurate and contextually relevant extended text $C_{t}^{samp}$. In our model, the decoder is implemented using a fully connected layer.

This form of representation allows the model to move beyond a fixed point when representing a class, enabling it to learn and express a neighborhood region that covers potential variations. Such flexibility is crucial under few-shot conditions, where limited samples are often insufficient to capture the full semantic space of a class. By introducing distribution modeling, the network is encouraged to learn a more generalizable representation, allowing class boundaries to better accommodate intra-class variability and inter-class similarity.

During training, our objective is to ensure that the joint latent representation $z_{t}^{joint}$ can accurately integrate key information from both the category text and the image support samples, thereby enabling a more effective representation of the true semantics of each class. To achieve this, we design a loss function that guides the model-generated extended class description $C_{t}^{samp}$ to remain semantically consistent with the original modality inputs while enhancing its cross-modal perception capability. Specifically, the loss function consists of two main components: (1) a reconstruction term, which measures whether the generated class description $C_{t}^{samp}$ reflects the semantics expressed by the fused textual and visual features, and (2) a regularization term, which enforces the stability and generalization of the latent representation space, ensuring consistency across different tasks. The overall optimization objective is formulated as follows:

(10) $$\begin{array}{cc} L_{res} & =E_{q(z_{t}^{joint}|C_{t}^{expand},P_{t}^{image})}\left[logp\left(C_{t}^{samp}|z_{t}^{joint}\right)\right]\\ & -D_{KL}(q\left(z_{t}^{joint}|C_{t}^{expand},P_{t}^{image}\right)\|p\left(z_{t}^{joint}\right)). \end{array}$$

The first term reflects the decoder’s ability to reconstruct the semantic text from the joint latent representation, ensuring that the generated extended class information incorporates both visual context and semantic accuracy. The second term serves as a regularization component, helping the model maintain structural stability in the latent space and reducing overfitting caused by limited samples. By minimizing this loss function through backpropagation, the model is iteratively optimized during training, enabling the generated class descriptions to more comprehensively integrate both image and text information. The resulting enhanced class representations are not only semantically richer but also more suitable for subsequent prototype construction and few-shot classification—especially in scenarios with incomplete modal information (e.g., blurred images) or insufficient semantics (e.g., underdeveloped class descriptions).

By using the backpropagation algorithm to minimize the KL divergence and reconstruction error, the model parameters are optimized. Through multiple training iterations, the parameters are fine-tuned so that the generated latent variables $z_{t}^{joint}$ can better capture the joint features of both the image and text information. Through the above process, we obtain the enhanced textual features $C_{i}^{samp}$.

#### 3.2.2. Multi-Stage Fusion Module

In multimodal few-shot classification, efficiently integrating heterogeneous modalities is a core challenge. While the previous section focused on enhancing text features, this section concentrates on achieving more effective cross-modal fusion based on these augmented representations. Text and images, as distinct encoding systems, inherently possess a ‘semantic gap,’ implying potentially very low mutual information between their raw feature representations. Traditional fusion methods, such as feature weighting or direct concatenation, often act as a ‘lossy channel’ because they fail to effectively bridge this gap, leading to information loss. Particularly in deep networks, due to the data processing inequality, information can only decrease or remain constant as it propagates layer by layer. This creates an ‘information bottleneck,’ limiting the discriminative power of the final representation. To address this issue, we propose a cross-modal hierarchical residual connection strategy. This approach aims to fully leverage the strengths of each modality for specific tasks and enhance the effectiveness of cross-modal feature interaction. Compared to traditional residual connections, our method optimizes the problem of information loss in deep networks by implementing a layer-by-layer residual connection mechanism, thereby improving the efficiency of information flow across different levels.

Specifically, we design a multi-stage multimodal fusion module, as illustrated in the lower part of Figure 1. This module consists of multiple hierarchical multimodal fusion units. Each fusion unit receives four inputs: two pre-stage features (Pre-image and Pre-text) and two current-stage features (Cur-image and Cur-text). The current-stage features are outputs from the previous stage, while the pre-stage features are set as the initial image features and the enhanced textual features. All fusion units share the same structure but do not share parameters, ensuring that features at different levels can fully learn cross-modal information. This hierarchical structure, combined with residual connections, is designed to build an efficient information flow. Residual connections provide a direct path for earlier (shallower) information to reach later (deeper) layers, ensuring the integrity of fundamental information. This allows the network to primarily learn the residual part (i.e., the incremental information), which is often easier to converge.

**Figure 2:**
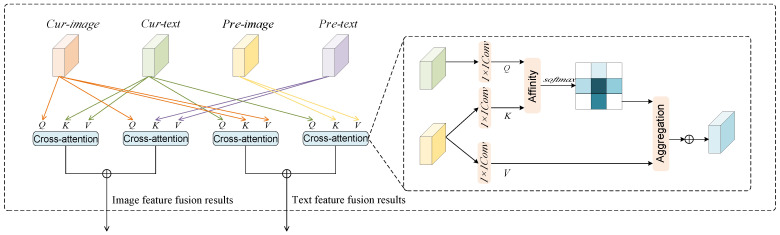
Cross-Modal Hierarchical Residual Connection Module. The module applies four cross-attention operations: (1) Cur-image as Q, Cur-text as K and V, aligning current image and text features; (2) Cur-image as Q, Pre-image as K and V, integrating historical visual context; (3) Cur-text as Q, Cur-image as K and V, refining text with visual cues; and (4) Cur-text as Q, Pre-text as K and V, incorporating residual information from the previous text layer. The right panel illustrates the cross-attention computation.

As shown in Figure 2, the multimodal fusion unit consists of four cross-attention mechanisms: two for computing the fusion between text and image within the current layer (i.e., the interaction of the current-stage features) and two for modeling cross-layer information transfer between the current and previous layers (i.e., the interaction of the pre-stage and current-stage features). When computing cross-attention in the current layer, the first cross-attention mechanism uses image features as the primary representation, where text features serve as the query and image features act as the key–value pairs. To enhance the flexibility and adaptability of the attention mechanism, we introduce a learnable parameter matrix to optimize the fusion process:

(11) $$Att_{p}\left(C_{t}^{samp}\left(l\right),P_{t}^{image}\left(l\right)\right)=\mathrm{softmax}\left(\frac{C_{t}^{samp}\left(l\right)W_{text}{\left(P_{t}^{image}\left(l\right)\right)}^{T}}{\sqrt{d_{k}}}\right)P_{t}^{image}\left(l\right),$$

where *W*_*text*_ represents the learnable weight matrix. By introducing only one learnable parameter matrix instead of fixed mappings or hard-coded weights, the model’s training process can capture the generality and potential non-linear relationships between cross-modal features. *d*_*k*_ refers to the dimension of attention. Elements with the subscript *l* are denoted as the current-stage features.

The second cross-attention mechanism then computes the fusion result with text features as the primary representation, where the image features serve as the query and the text features are used as key–value pairs:

(12) $$Att_{c}\left(C_{t}^{samp}\left(l\right),P_{t}^{image}\left(l\right)\right)=\mathrm{softmax}\left(\frac{P_{t}^{image}\left(l\right)W_{image}{\left(C_{t}^{samp}\left(l\right)\right)}^{T}}{\sqrt{d_{k}}}\right)C_{t}^{samp}\left(l\right),$$

where *W*_*image*_ is the learnable weight.

In cross-modal learning, the interaction between image and text is crucial. To enhance the model’s understanding and representation of both modalities, we not only compute the interaction between the text and image at the current layer but also incorporate the text and image features from the previous layer into the current layer. This helps further improve the flow of information and semantic alignment. In the cross-modal hierarchical residual connection, the third cross-attention mechanism computes the cross-attention between the text features $C_{t}^{samp}\left(l\right)$ and the image features $P_{t}^{image}\left(l-1\right)$. Elements with subscript (*l* − 1) are denoted as the pre-stage features. Specifically, we use the following formula to compute the cross-attention from text to image:

(13) $$\begin{array}{cc} Att_{t2i}(C_{t}^{samp}\left(l\right),\; & P_{t}^{image}\left(l-1\right))\\ & =\mathrm{softmax}\left(\frac{C_{t}^{samp}\left(l\right)W_{t2i}{\left(P_{t}^{image}\left(l-1\right)\right)}^{T}}{\sqrt{d_{k}}}\right)P_{t}^{image}\left(l-1\right), \end{array}$$

where *Att*_*t*2*i*_ is the weight matrix used to control the interaction between the text and image features. In this process, the text features, acting as queries, are used to associate with the image features from the previous layer. Similarly, the fourth cross-attention mechanism computes the cross-attention between the image features and the text features from the previous layer:

(14) $$\begin{array}{cc} Att_{i2t}(C_{t}^{samp}\left(l-1\right),\; & P_{t}^{image}\left(l\right))\\ & =\mathrm{softmax}\left(\frac{P_{t}^{image}\left(l\right)W_{i2t}{\left(C_{t}^{samp}\left(l-1\right)\right)}^{T}}{\sqrt{d_{k}}}\right)C_{t}^{samp}\left(l-1\right), \end{array}$$

where *W*_*i*2*t*_ is the weight matrix that controls the interaction between the image and text features.

To further enhance the flexibility of cross-modal fusion path selection, we propose a path self-aware routing mechanism based on cross-attention representations. From an information theory perspective, the goal of multimodal fusion is to minimize uncertainty while integrating information from different sources. Traditional methods typically generate fusion weights from the entire support set, a relatively coarse approach to information processing that struggles to achieve optimal information filtering and fusion for specific tasks. Our method introduces a more refined dynamic routing process. In information theory, entropy measures the uncertainty or disorder of information. To make better decisions with complex cross-modal data, we need to reduce the entropy in the decision-making process. This method directly utilizes the outputs of multiple cross-attention paths at the current layer as input for the dynamic routing process, which is essentially a process of information filtering and refinement. This allows the model to automatically assign weights to different information paths according to the specific needs of the task. This enables the model to more intelligently determine which information paths are more important for the current task, thereby reducing decision uncertainty and achieving more fine-grained task-specific adaptation.

To this end, we have designed a task encoder based on a multi-layer attention architecture. Given the four outputs $Att=\left[Att_{p},Att_{c},Att_{t2i},Att_{i2t}\right]$, the encoder processes each cross-attention output *Att*_*i*_ to extract high-level semantic information. Specifically, each path output is first passed through a linear transformation and an activation function to map it into a higher-level semantic representation space. This process can be understood as encoding and compressing the original information to extract its most core and task-relevant features, thereby providing high-quality input for the subsequent generation of path weights:

(15) $$h_{i}=\sigma \left(W_{1}Att_{i}+b_{1}\right),$$

where *W*_1_ and *b*_1_ are learnable parameters and *σ* denotes the activation function. Subsequently, we average the semantic vectors *h*_*i*_ from all paths to obtain a global task representation *h*_*avg*_. Then, we compute the importance score *α*_*i*_ for each path using a dot-product attention mechanism:

(16) $$\alpha_{i}=\frac{exp\left(\left(h_{i}W_{q}\right){\left(h_{avg}W_{k}\right)}^{T}/\sqrt{d_{k}}\right)}{\sum_{j}exp\left(\left(h_{j}W_{q}\right){\left(h_{avg}W_{k}\right)}^{T}/\sqrt{d_{k}}\right)},$$

where *W*_*q*_ and *W*_*k*_ are learnable parameters. The attention scores are further refined through a projection layer to generate the final fusion path weight vector:

(17) $$\gamma_{i}^{\left(T\right)}=\mathrm{softmax}\left(W_{2}{\left[\alpha_{1},\alpha_{2},\alpha_{3},\alpha_{4}\right]}^{T}+b_{2}+\alpha_{i}\right),$$

where *W*_2_ and *b*_2_ are learnable parameters. Finally, the outputs of the four cross-attention paths are combined through a weighted summation using the computed weights. This process effectively reduces modal redundancy, enhances semantic consistency, and ensures that the generated class prototype representations are more context-aware, making them well-suited for prototype matching in few-shot classifiers. The final fused representation is then produced through a multi-layer perceptron:

(18) $$Att_{f}=MLP\left(\sum_{i=1}^{4}\gamma_{i}^{\left(T\right)}Att_{i}\right).$$

#### 3.2.3. Model Training Process

In the previous chapter, we provided a detailed explanation of how to construct reconstructed prototypes using the proposed method. This chapter will focus on the training process of the model based on this prototype construction approach. In few-shot learning tasks, the model is trained on a Base Set, aiming to learn how to effectively classify new samples using only a limited number of labeled examples. For each category, its sample distribution can be represented by an aggregated center, known as the category prototype *C*. The computation of the prototype is as follows: Given a support set *S*_*n*_, which contains all samples (*x*_*i*_, *y*_*i*_) ∈ *S*_*n*_ belonging to class *n*, the class prototype *C*^*n*^ is defined as the mean vector of the embedded representations:

(19) $$C^{n}=\frac{1}{\left|S_{n}\right|}\sum_{\left(x_{i},y_{i}\in S_{n}\right)}f_{\phi }\left(x_{i}\right),$$

where *f*_*ϕ*_ is a neural network used to extract embedding representations, mapping the input *x*_*i*_ into the embedding space. *C*^*n*^ denotes the prototype of class *n*, representing the center of this class in the embedding space. $\left|S_{n}\right|$ indicates the number of support samples belonging to class *n*.

In this study, we construct reconstructed prototypes using the multimodal fusion results obtained in the previous section and use them as the foundation for classification in the testing phase. During training, we optimize the alignment between the reconstructed prototype and the true prototype to better match the category distribution, thereby improving the model’s classification performance. Specifically, we optimize by minimizing the distance between the reconstructed prototype *Att*_*f*_ and the true prototype *C*. To achieve this, we define a loss function that constrains and optimizes this distance, thereby enhancing the model’s generalization ability for few-shot classification tasks:

(20) $$L_{align}=\sum_{n=1}^{N}\sum_{\left(x_{i},y_{i}\in S_{n}\right)}{∥Att_{f}^{i}-C^{n}∥}_{2},$$

where *N* is the number of categories in the Base Set. Combining the loss from the multi-level fusion method with name expansion and image feature augmentation, the overall loss function during training is

(21) $$L=L_{align}+L_{res}.$$

#### 3.2.4. Model Testing Phase

**Figure 3:**
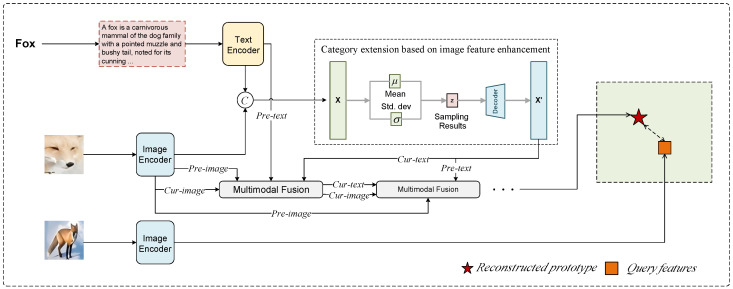
Flowchart of the Testing Phase. During the testing phase, all parameters remain fixed. The model first constructs prototypes based on the input support set. Then, it extracts features of the query set using the visual encoder and compares them with the reconstructed prototypes to determine the query set’s categories.

In the training phase (Section 3.2.3), the model learns to construct reconstructed prototypes and generate more discriminative category representations using multimodal information. During the testing phase of few-shot learning, the model’s parameters are frozen and no longer updated. Specifically, during the testing phase, the support set is first used to compute the reconstructed prototype for each category, ensuring it adequately represents the feature distribution of that category. Then, for each query sample, the model calculates similarity scores between the query and all reconstructed prototypes. Finally, the query sample is classified into the category corresponding to the reconstructed prototype with the smallest distance. The entire process is illustrated in Figure 3. This testing strategy ensures that the model can effectively generalize to new categories under limited sample conditions while maintaining stable classification performance. The following outlines the process of computing the reconstructed prototype:

(22) $$C_{res^{n}}=\frac{1}{M}\sum_{i=1}^{M}Att_{f}^{i},$$

where *M* represents the number of categories *n* in the support set. Next, for a given query sample *q*, we first extract its image and text features and then obtain the fused feature representation $Att_{f}^{q}$ of the query sample through multimodal fusion. We calculate the distance between the query sample’s features and the reconstructed prototype of each category as follows:

(23) $$d\left(Att_{f}^{q},C_{res}^{n}\right)=\|Att_{f}^{q}-C_{res}^{n}{\|}_{2}.$$

Based on the calculated distances between the query sample and each category’s reconstructed prototype, we assign the query sample to the category with the smallest distance to its reconstructed prototype. Specifically, the classification result of the query sample *q* is

(24) $$y_{q}=argmin_{n}\left(d\left(Att_{f}^{q},C_{res}^{n}\right)\right).$$

Through this process, the model establishes robust category representations in the feature space based on the reconstructed prototype classification strategy. This enables the model to accurately classify unseen categories by relying on the learned metric space without requiring additional updates to the model parameters. This approach not only effectively reduces dependence on large amounts of labeled data but also significantly enhances the generalization ability of few-shot learning. It enables the model to quickly adapt to new classification tasks while ensuring stable and robust classification performance.

## 4. Experiments

### 4.1. Dataset Settings

This study conducts experiments on both public and self-constructed datasets. The public datasets include CIFAR-FS [27] and FC100 [28], both derived from CIFAR-100. CIFAR-FS uses a random partition strategy and contains 64 training classes, 16 validation classes, and 20 test classes, aiming to evaluate the model’s classification ability under few-shot conditions. And FC100 introduces a unique superclass division method, where the 100 categories are divided into multiple superclasses. The training set contains 12 superclasses, totaling 60 classes, while the validation and test sets each contain 4 superclasses, totaling 20 classes. This superclass division method increases the task difficulty, requiring the model not only to recognize individual categories but also to handle semantic relationships between categories. This raises the model’s requirements for generalization ability and cross-modal learning capability. Through experiments on these two public datasets, this study comprehensively evaluates the proposed method’s performance under different task settings and validates its effectiveness in few-shot learning tasks.

In our self-constructed dataset for this study, we collaborated with Yantai Yuhuangding Hospital to collect multimodal medical data from 255 pathologically confirmed breast cancer patients. All data acquisition was conducted under hospital approval and strictly adhered to protocols of informed consent and patient privacy protection. The inclusion criteria required patients to have a histopathological diagnosis of breast cancer, complete clinical records, and available imaging and pathological slide data, while exclusion criteria included patients with incomplete data, ambiguous pathological diagnoses, or poor-quality imaging unsuitable for analysis. Specifically, the medical imaging data include magnetic resonance imaging (MRI) and ultrasound scans, acquired by radiology experts following standard clinical procedures; pathological tissue slide images, which were prepared and digitized by professional pathologists during routine diagnosis; and clinical textual data, encompassing demographic information, laboratory test results, tumor size and staging, as well as molecular subtype annotations. To ensure annotation consistency, all pathological and molecular subtype labels were independently reviewed by at least two senior pathologists and radiologists, with discrepancies resolved through consensus discussion. Leveraging the clinical resources and expertise of Yuhuangding Hospital, this dataset ensures high clinical authenticity and representativeness, thereby providing a solid foundation for research on multimodal learning and molecular subtype classification in breast cancer.

We classified all samples based on the clinical indicators recorded in the text, ultimately dividing the entire dataset into 80 categories. These categories represent the differences in clinical characteristics of breast cancer patients, covering various tumor types, pathological features, and molecular subtypes. In the dataset division, the training set contains 50 categories, the validation set contains 10 categories, and the test set contains 20 categories. This division ensures the diversity of the dataset while meeting the requirement for non-overlapping categories in few-shot learning. Although the average number of samples per category is relatively small (approximately three cases), this design is intentional: it reflects the clinical heterogeneity of breast cancer, where many subtypes and pathological combinations occur at low frequency in practice. Therefore, our dataset mimics real-world diagnostic challenges with long-tail distributions, making it particularly suitable for evaluating few-shot learning models under clinically realistic conditions.

﻿In clinical practice, breast cancer is not a single, uniform disease but a highly heterogeneous condition composed of various biological subtypes, pathological types, and clinical stages. Dividing patients into 80 fine-grained categories based on key clinical indicators such as age, tumor size, T stage, and molecular subtype is essentially equivalent to simulating 80 specific patient types or diagnostic labels in real-world settings. Examples include middle-aged patients with HER2-positive tumors, T2 stage, and tumor diameter greater than 2 cm; young patients with triple-negative breast cancer, early-stage disease but high Ki-67 index; or elderly patients with ER-positive, Luminal A-type tumors and ductal carcinoma pathology. This method of classification goes beyond the simple benign/malignant or four-subtype models, aligning closely with clinical stratification strategies. Each category represents a potential branch of diagnostic or treatment decision-making, making it more suitable for scenarios such as individual risk prediction, treatment response grouping, and precision follow-up stratification.

### 4.2. Implementation Details

In our experiments, we adopt a pre-trained Swin-T [31] as the visual encoder to fully exploit the hierarchical representation capability of images, while the text modality uses the text encoder from ViT-B/16 CLIP [32], with an output dimension of 512 to ensure rich and compact semantic representations. The training procedure is conducted in two stages: in the first stage, the pretraining of Swin-T follows the same strategy as FewTURE [15] to ensure fair comparison under the same experimental settings; in the second stage, the entire network is trained end-to-end for 50 epochs with a batch size of 128, using the Adam optimizer with a fixed learning rate of 1 × 10^−4^. The text encoder and the image encoder are trained simultaneously. This two-stage training strategy maintains the stability of visual features while effectively facilitating cross-modal alignment. During evaluation, the model is tested under standard five-way one-shot and five-way five-shot settings, with multiple tasks constructed for each setting to cover different combinations of classes, thereby comprehensively validating the robustness and generalization performance of the proposed method.

### 4.3. Comparison Experiments

To comprehensively evaluate the effectiveness of the proposed method in multimodal few-shot learning, this study compared it with several advanced methods in the field of few-shot learning. These comparison methods cover various few-shot learning strategies, including metric-based learning, meta-learning, and cross-modal learning models. We used the publicly available datasets CIFAR-FS, FC100, and our self-constructed breast cancer dataset to validate the performance of our method under different task settings.

The comparison methods include ProtoNet [19], TADAM [33], MetaOptNet [34], MABAS [35], RFS [17], SUN [36], FewTURE [15], Meta-AdaM [37], SP-CLIP [38], and SemFew [23]. MABAS combines a memory-augmented attention mechanism, effectively improving classification performance through memory enhancement and attention weighting. RFS builds a relational graph model through relation learning, capturing similarities between samples and effectively propagating information in few-shot scenarios. SUN uses the ViT model and employs self-supervised learning to effectively encode image features, thereby improving classification accuracy. FewTURE proposes a task-aware self-supervised learning strategy, utilizing the Swin Transformer structure to enhance feature embeddings for few-shot learning. Meta-AdaM, based on meta-learning, uses an adaptive memory module that dynamically adjusts the memory mechanism to enhance classification performance in few-shot learning. SP-CLIP combines cross-modal pretraining with image and text features for few-shot learning, effectively handling visual and linguistic information using the Visformer-T model, with its image encoder initialized from CLIP’s ViT-B/16 and fine-tuned for 30 epochs using AdamW. SemFew leverages semantic augmentation by enriching class labels with external knowledge and mapping them into the same feature space as images, where ResNet-12 is used as the backbone, trained for 60 epochs with SGD, under the standard 5-way one/five-shot episodic training setting.

First of all, as shown in Table 1, on the CIFAR-FS dataset, our method outperformed all comparison methods in both the five-way one-shot and five-way five-shot settings. Notably, in the five-way one-shot setting, our method achieved a classification accuracy of 86.45, outperforming SP-CLIP and SUN by 4% and 8%, respectively. This demonstrates the significant advantage of our method in few-shot settings. This improvement can be attributed to the multimodal information fusion in our approach, particularly the effective combination of category name expansion and image features. This enables the model to extract richer semantic and visual features from the limited labeled samples, thereby enhancing its classification capability.

On the FC100 dataset, as shown in Table 1, our method also performed excellently, achieving classification accuracies of 56.14 and 67.21 in the five-way one-shot and five-way five-shot settings, respectively, outperforming methods like SP-CLIP and FewTURE. Compared to MABAS and RFS, our method improved by 14%. This indicates that our cross-modal feature fusion strategy can effectively enhance the model’s ability to recognize few-shot categories, especially when there are significant semantic differences between categories. On our structured cancer dataset, as shown in Table 2, the classification results of two multimodal small sample tasks, MRI and text, pathology and text, are presented. In the five-way one-shot setting, our method achieved a classification accuracy of 56.87% and 52.49%. In the five-way five-shot setting, it reached 62.53% and 59.56%. Compared to other methods, such as ProtoNet, our method improved by 3.00%. This shows that, in medical image tasks, our method effectively combines image and text information, providing richer and more accurate category information when handling complex medical images and clinical text data.

### 4.4. Ablation Study

#### 4.4.1. Effectiveness of Image Feature Augmentation

This section aims to evaluate the enhancement effect of image features on category name expansion. By introducing image features, we hope to enable the model to better understand the actual visual representation of categories and improve the model’s classification ability. To this end, two models are compared in this experiment: (1) Text Expansion Only: This model uses only the expanded category text generated by a large language model as input, without incorporating image features. (2) Text Expansion + Image Features: This model combines image features with the expanded category text, enhancing the category expansion text through conditional generation. By comparing these two models, we aim to explore the supplementary role of image features in category text expansion, particularly in data-scarce scenarios, to determine whether it can significantly improve the model’s generalization ability.

We conduct classification tasks on both public datasets (CIFAR-FS, FC100) and our self-built dataset, comparing the classification performance of the two models under different task settings. For each setting, we evaluate the model performance by calculating the Top-1 accuracy. The experimental results are shown in Table 3. In the experimental results, we found that on both the CIFAR-FS and FC100 public datasets, the fusion model that combines image features with expanded category texts showed a significant improvement in Top-1 accuracy compared to the model that only uses text expansion. Particularly in the five-way one-shot task, the inclusion of image features notably enhanced classification accuracy, demonstrating the supportive role of image information for the model. On the self-constructed breast cancer dataset, the supplementation of image features showed particularly strong performance in medical image classification tasks. In both the five-way one-shot and five-way five-shot tasks, incorporating image features for enhancement leads to significant improvements. Due to the complexity of medical images, relying solely on text expansion may not be sufficient to capture the visual details of the images. The introduction of image features allowed the model to better understand the image content, thereby further improving the model’s classification accuracy.

**Figure 4:**
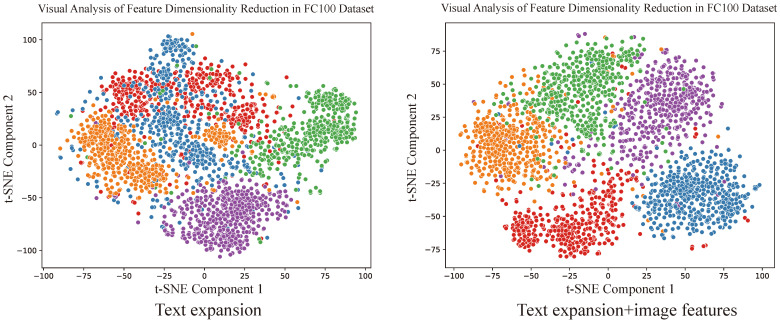
Visual analysis of image feature supplementation. Note that different colors represent different classes.

To further validate the supplemental role of image features, we conducted a qualitative analysis on certain categories from the FC100 dataset. Using t-SNE dimensionality reduction for visualization, we reduced the feature representations of the model that combines text expansion and image feature fusion and observed the distribution of categories. The experimental results are shown in Figure 4. When only text expansion was used, the feature distribution of categories was more scattered, with lower distinguishability between the features, and the distance between categories was smaller. In contrast, when image features were incorporated, the feature distribution of categories showed a significant change, with increased distances between categories and more pronounced clustering effects.

Additionally, we evaluated the robustness of the image feature enhancement module against noisy extended text and verified how image features improve the quality of text expansion under noise interference. The study focused on two typical types of noise: random replacement (replacing 20% of the keywords in the expanded text with irrelevant words) and truncation (retaining only the first 50% of the expanded text), and was tested on the CIFAR-FS dataset. The experiment compares the baseline-text model, which uses only text expansion, with the baseline-image model, which incorporates image feature enhancement, to analyze the image feature’s robustness in noisy environments. The five-way one-shot classification task is used, and the improvement in classification accuracy (ΔAcc) quantifies the performance gain of the baseline-image model compared to the baseline-text model under noise conditions. The results are shown in Table 4. The experimental results show that under noise-free conditions, the baseline-image model achieves an accuracy of 86.45%, a 2.33% improvement over the baseline-text model (84.12%). Under random replacement noise, the accuracy of baseline-image drops to 83.75%, while baseline-text falls to 81.20%, resulting in a ΔAcc of 2.55%. In the truncation noise setting, baseline-image reaches 84.20%, compared to 82.10% for baseline-text, yielding a ΔAcc of 2.10%. These results indicate that image feature enhancement significantly improves the robustness of text expansion, especially under random replacement, by providing additional visual constraints that help the model better capture category semantics.

#### 4.4.2. Learnable Category Embedding Distribution Modeled

**Figure 5:**
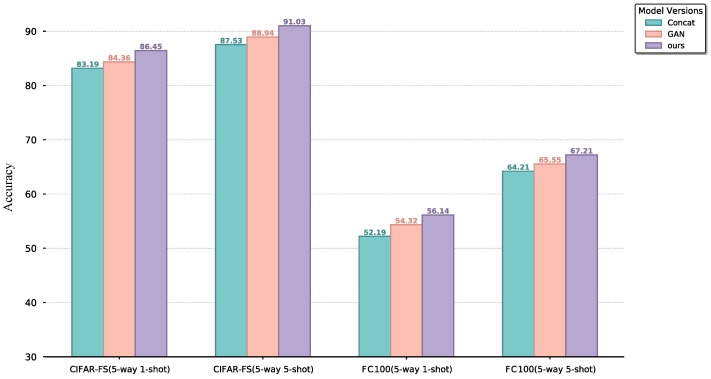
Ablation Study of Learnable Category Embedding Distribution Modeled.

This section aims to evaluate the effectiveness of the learnable category embedding distribution modeled $z_{t}^{joint}$ in few-shot learning tasks, particularly its role in multimodal feature fusion. The $z_{t}^{joint}$ integrates image features into the text augmentation process to enhance category representation. To comprehensively assess its contribution, we design three comparative experiments. The full model retains the $z_{t}^{joint}$. In contrast, the direct concatenation method removes the $z_{t}^{joint}$ and directly concatenates text and image features before inputting them into the decoder. The adversarial generation method replaces the $z_{t}^{joint}$ with a Generative Adversarial Network (GAN) to produce joint features. Experiments are conducted on the CIFAR-FS and FC100 datasets under five-way one-shot and five-way five-shot tasks, using classification accuracy as the evaluation metric. The experimental results are illustrated in Figure 5.

The experimental results demonstrate that the $z_{t}^{joint}$ consistently improves classification performance across datasets. On the CIFAR-FS dataset, the full model achieves 86.45% accuracy on the five-way one-shot task, which further improves to 91.03% on the five-way five-shot task. Compared with the direct concatenation and adversarial generation methods, the $z_{t}^{joint}$ shows a more significant performance boost. This trend indicates that the $z_{t}^{joint}$ not only enhances feature fusion under scarce data but also effectively suppresses modality conflicts as more data becomes available. On the FC100 dataset, where high semantic overlap between categories poses greater challenges to generalization, the full model achieves the highest accuracy on the five-way one-shot task. This indicates that the $z_{t}^{joint}$ effectively performs cross-modal feature adaptation via variational inference, mitigating class confusion and enhancing discriminative power in complex scenarios. The experiment shows that direct concatenation is sensitive to local noise, and adversarial generation suffers from distribution shifts due to training instability. In contrast, the $z_{t}^{joint}$ significantly enhances classification performance by modeling the probability of latent variables, enabling the deep fusion of textual descriptions and visual features. This process results in more discriminative class prototypes, improving the model’s ability to generalize and distinguish between categories.

**Figure 6:**
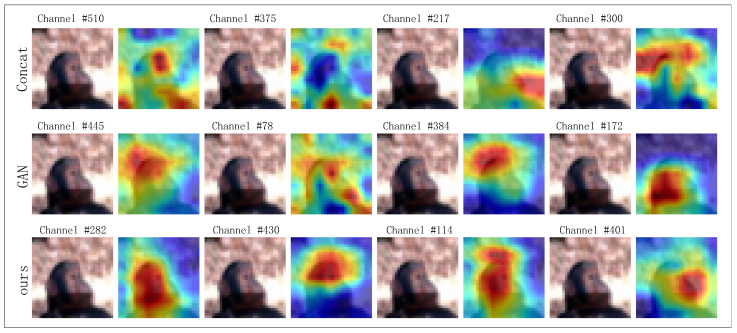
Channel response heatmap in the ablation experiment of Learnable Category Embedding Distribution Modeled.

At the same time, we performed a visualization analysis of the first-layer multimodal fusion process using channel response heatmaps in the five-way one-shot task of the CIFAR-FS dataset. We selected the top four most active channels to examine the $z_{t}^{joint}$’s role in subsequent cross-modal feature fusion. The experimental results, shown in Figure 6, indicate that the high-response channels of the complete model (including the $z_{t}^{joint}$) are primarily focused on key semantic areas of the target category, demonstrating its ability to accurately capture discriminative features for classification. In contrast, the high-response areas of the direct concatenation method are more scattered, with some channels’ activation signals covering the background, leading to reduced focus on the category itself. Furthermore, the high-response channels of the adversarial generation method show significant instability, lacking sufficient discriminative power for fine-grained classification, which impacts the model’s classification decisions.

#### 4.4.3. Cross-Modal Hierarchical Residual Connections

This section verifies the enhancing effect of cross-modal hierarchical residual connections on information flow and semantic alignment. We aim to analyze through experiments whether cross-modal hierarchical residual connections can effectively prevent information loss and promote better alignment between image and text features. We designed two models for comparison: (1) No Residual Connection: This model removes the cross-modal residual connections and uses only a single-layer cross-attention mechanism for the fusion of image and text features. (2) Hierarchical Residual Connection: This model uses cross-modal hierarchical residual connections, where residual connections ensure that the output of each layer retains the original information during the feature fusion process. This experimental setup helps us to deeply understand the impact of cross-modal residual connections on model performance. The experimental results are shown in Table 5.

The experimental results show that the model with hierarchical residual connections consistently outperforms the model without residual connections in terms of classification accuracy on the CIFAR-FS and FC100 datasets. The advantage of hierarchical residual connections is particularly evident in the five-way one-shot task of FC100 and five-way five-shot task of CIFAR-FS. Due to the extreme scarcity of data in few-shot tasks, the model relies heavily on efficiently utilizing the limited data. The hierarchical residual connection helps the model maintain and enhance the features at each layer during the information flow, leading to stronger performance in the classification task.

**Figure 7:**
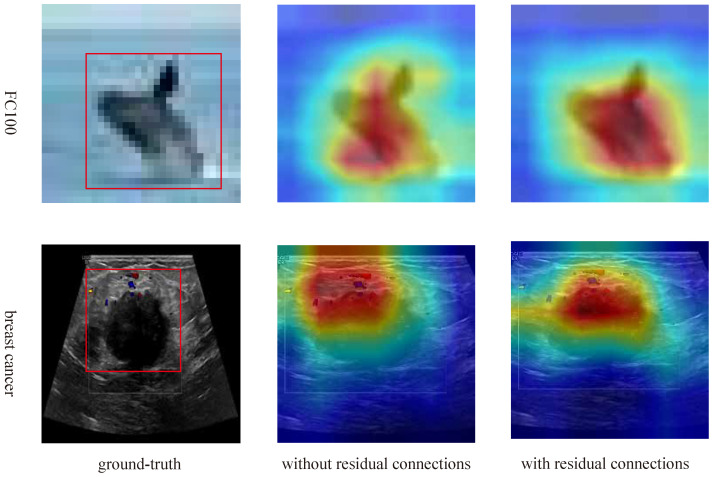
Grad-CAM visualization analysis of cross-modal hierarchical residual connections.

To further validate the effect of hierarchical residual connections, we applied Grad-CAM for visual analysis of the model. The experimental results are shown in Figure 7. Grad-CAM generates heatmaps by calculating the gradients in the convolutional neural network, showing which regions of the image the model focuses on during prediction. We applied Grad-CAM to the image portion and combined it with the text features for analysis to explore the interaction between the image and text in the model’s decision-making process. On the FC100 and breast cancer datasets, when performing image classification, the model without residual connections is able to focus on some important regions of the image. However, it tends to pay less attention to certain detailed features, especially in tasks where categories are similar or visually overlapping. In such cases, the model often fails to capture subtle differences in the image. In contrast, the model with hierarchical residual connections, as shown by Grad-CAM, exhibits clearer and more accurate focus areas.

**Figure 8:**
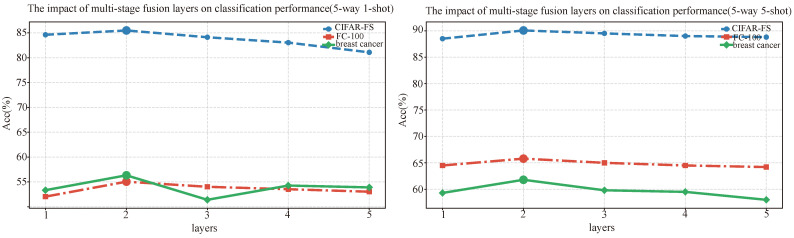
The impact of the number of layers in the multi-stage fusion module on model performance.

We also explored the impact of the number of layers in the multi-stage fusion module on model performance, aiming to analyze the role of different fusion depths in cross-modal feature interaction and semantic alignment. We tested models with fusion layers ranging from 1 to 5 on the CIFAR-FS, FC100, and breast cancer datasets to validate the importance of hierarchical fusion in few-shot learning tasks. Figure 8 shows the classification accuracy line charts for different fusion layer numbers across the three datasets. The experimental results indicate that the two-layer fusion structure achieves the best performance in the five-way one-shot task, while the five-layer model shows a 1.2% drop in classification accuracy on the medical dataset. The two-layer residual fusion achieves a better balance between capturing complementary multimodal cues and avoiding redundant information. Specifically, the first layer primarily aligns low-level representations across modalities, while the second layer further integrates semantic information to enhance discriminative features. Adding more layers, however, tends to amplify noise or redundant correlations, leading to overfitting and reduced generalization, especially in small-sample medical scenarios. This suggests that excessively deep fusion structures may lead to feature overfitting or information redundancy, which affects generalization. The results highlight the need to balance fusion depth between information transmission efficiency and model complexity to avoid both shallow structure’s inadequate expression and deep structure’s feature shift issues.

**Figure 9:**
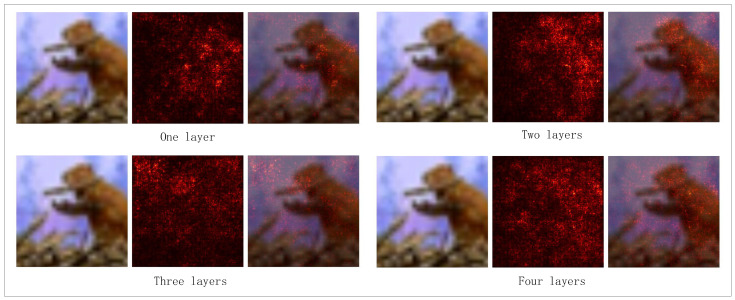
Saliency maps corresponding to different layers of multimodal fusion modules.

We also plotted the saliency maps of the visual encoder for models with different layer counts to compare their feature focusing capabilities. The experimental results are shown in Figure 9. In the CIFAR-FS classification task, the two-layer model showed clearer activation in the key regions of the target category compared to the one-layer model, demonstrating stronger feature selection ability. However, the three-layer and four-layer models exhibited a diffusion trend in the activation areas, with some attention scattered to the background, indicating that deeper networks might weaken feature discrimination and cause the model to focus on irrelevant regions. Overall, the results and visual analysis from the two-layer fusion structure across different datasets demonstrate its significant advantages in cross-modal feature alignment, local feature enhancement, and improved classification performance. Moderate hierarchical fusion ensures sufficient interaction of cross-modal information, while overly shallow or deep fusion structures may lead to insufficient information expression or feature dispersion, negatively impacting the model’s final performance.

## 5. Conclusions

This paper proposes an innovative few-shot learning method that combines category name expansion with image feature augmentation. By incorporating extended text generated by large language models and image features into the traditional metric learning framework, we enable the model to gain a more comprehensive understanding of both the semantic and visual characteristics of categories, significantly improving classification performance under scarce data conditions. Through a series of ablation experiments, we validated the role of image features in enhancing category expansion texts. Additionally, we employed cross-modal hierarchical residual connections to optimize information flow and semantic alignment, further improving the effectiveness of multimodal fusion. Experimental results show that the proposed method outperforms several advanced comparison methods on the CIFAR-FS, FC100, and medical image datasets, demonstrating its exceptional performance in few-shot learning tasks. In particular, it effectively enhances the model’s learning and generalization abilities, especially in scenarios where there are significant semantic differences between categories. In future work, we will further optimize the multimodal feature fusion method. Although the attention-based fusion method used in this study has significantly improved performance, there is still room for further enhancement, particularly in dynamically adjusting the importance of image and text information. We aim to explore more feature fusion strategies based on self-attention mechanisms or graph neural networks to further improve the efficiency and effectiveness of multimodal fusion.

## Figures and Tables

**Table 1:** Comparison with state-of-the-art methods on the CIFAR-FS and FC100 datasets.

Method	CIFAR-FS		FC100	
	5-Way 1-Shot	5-Way 5-Shot	5-Way 1-Shot	5-Way 5-Shot
ProtoNet	72.20 ± 0.70	83.50 ± 0.50	41.54 ± 0.76	57.08 ± 0.76
TADAM	-	-	40.10 ± 0.40	56.10 ± 0.40
MetaOptNet	72.80 ± 0.70	84.30 ± 0.50	47.20 ± 0.60	55.50 ± 0.60
MABAS	73.51 ± 0.92	85.65 ± 0.65	42.31 ± 0.75	58.16 ± 0.78
RFS	71.50 ± 0.80	86.00 ± 0.50	42.60 ± 0.70	59.10 ± 0.60
SUN	78.37 ± 0.46	88.84 ± 0.32	-	-
FewTURE	77.76 ± 0.81	88.90 ± 0.59	47.68 ± 0.78	63.81 ± 0.75
Meta-AdaM	-	-	41.12 ± 0.49	56.14 ± 0.49
SP-CLIP	82.18 ± 0.40	88.24 ± 0.32	48.53 ± 0.38	61.55 ± 0.41
SemFew	84.34 ± 0.67	89.11 ± 0.54	54.27 ± 0.77	65.02 ± 0.72
ours	86.45 ± 0.56	91.03 ± 0.61	56.14 ± 0.76	67.21 ± 0.75

**Table 2:** Comparison with state-of-the-art methods on the breast cancer dataset.

Method	MRI + Text		Pathology + Text	
	5-Way 1-Shot	5-Way 5-Shot	5-Way 1-Shot	5-Way 5-Shot
ProtoNet	52.43 ± 0.56	57.12 ± 0.64	48.32 ± 0.61	54.77 ± 0.58
MetaOptNet	53.12 ± 0.61	59.05 ± 0.70	49.89 ± 0.67	56.23 ± 0.62
MABAS	54.08 ± 0.63	60.21 ± 0.72	50.16 ± 0.66	57.34 ± 0.68
RFS	55.00 ± 0.62	60.55 ± 0.73	51.03 ± 0.69	58.10 ± 0.65
SUN	54.70 ± 0.65	59.85 ± 0.71	50.78 ± 0.70	57.98 ± 0.64
FewTURE	55.56 ± 0.64	61.24 ± 0.69	51.21 ± 0.68	58.40 ± 0.66
SemFew	55.12 ± 0.63	60.90 ± 0.72	50.50 ± 0.67	57.89 ± 0.69
ours	56.87 ± 0.54	62.53 ± 0.66	52.49 ± 0.72	59.56 ± 0.71

**Table 3:** Ablation experiment on image feature supplementation.

Method	CIFAR-FS		FC100		Breast Cancer (MRI + Text)	
	5-Way 1-Shot	5-Way 5-Shot	5-Way 1-Shot	5-Way 5-Shot	5-Way 1-Shot	5-Way 5-Shot
Without image	83.16 ± 0.58	89.50 ± 0.59	53.21 ± 0.75	64.95 ± 0.77	53.87 ± 0.52	60.87 ± 0.65
With image	86.45 ± 0.56	91.03 ± 0.61	56.14 ± 0.76	67.21 ± 0.75	56.87 ± 0.54	62.53 ± 0.66

**Table 4:** Ablation experiment on image feature supplementation under noise situation.

Noise Type	CIFAR-FS		
	Baseline-Text	Baseline-Image	ΔAcc
No Noise	84.12 ± 0.58	86.45 ± 0.56	+2.33
Random Replacement (20%)	81.20 ± 0.72	83.75 ± 0.65	+2.55
Truncation (50%)	82.10 ± 0.70	84.20 ± 0.68	+2.10

**Table 5:** Ablation experiment on cross-modal hierarchical residual connections.

Method	CIFAR-FS		FC100	
	5-Way 1-Shot	5-Way 5-Shot	5-Way 1-Shot	5-Way 5-Shot
without residual connections	84.23 ± 0.57	88.50 ± 0.60	53.95 ± 0.77	64.44 ± 0.74
with residual connections	86.45 ± 0.56	91.03 ± 0.61	56.14 ± 0.76	67.21 ± 0.75

## Data Availability

These data were derived from the following resources available in the public domain: [In Proceedings of the 905 Twenty-First International Conference on Artificial Intelligence and Statistics] [https://proceedings.mlr.press/v84/], accessed on 23 July 2025.
